# Comparing regional models of congenital bleeding disorders: preliminary steps in the Italian context

**DOI:** 10.1186/s13104-017-2552-6

**Published:** 2017-06-26

**Authors:** Sabina Nuti, Chiara Seghieri, Francesco Niccolai, Federica Vasta, Giuliano Grazzini

**Affiliations:** 1 Laboratorio Management e Sanità, Istituto di Management, Scuola Superiore Sant’Anna, Piazza Martiri della Libertà, 33, 56125 Pisa, Italy; 2 National Blood Centre, National Institute of Health, Rome, Italy

**Keywords:** Congenital bleeding disorders, Rare diseases management, Healthcare regionalization

## Abstract

**Background:**

Among these diseases, congenital bleeding disorders (CBD) represent a significant societal burden in terms of high morbidity costs and health outcomes. In Italy, the organization and provision of health care is a regional responsibility and regions must assure equity and quality to all their residents. This is also true for CBD care which is provided by 54 multidisciplinary Hemophilia Treatment Centers (HTCs) distributed among the regions. With the present study, we intend to stimulate a debate on the effect that the decentralization process have in the delivery of services to CBD patients across Italy.

**Methods:**

The available comparable measures of caseloads per center and interregional patient mobility, as proxies of quality and responsiveness of the regional network of HTCs, were first analyzed for the using data from the Italian Hemophilia Centers Association for the year 2012.

**Results:**

Nine thousand one hundred and thirty four Italian residents with CBD received care in at least one of the Italian HTC in 2012. Preliminary findings suggested room for improvement in health care delivery for CBD patients. In 2012, 16 HTCs out of 51 (31.4%) treated a number of patients under the minimum requirement for treatment center accreditation (10 severe patients). Moreover, data on interregional patient mobility highlighted differences in the ability of each region to retain its own residents or to attract residents from other regions.

**Conclusions:**

Preliminary study results showed significant disparities among regions in terms of volumes and mobility of residents with CBDs that cannot be completely explained by the different geographical characteristics. Therefore, the central government should consider taking concrete measures to bridge the gap between regions to assure access to quality care for all individuals with CBD independently from where they live and therefore to move toward a more integrated and homogeneous national network of care centers. Typology of disease, patients’ needs, and cost for outcomes, should have high priority on the political agenda. For CBD patients, even in a federal healthcare system, the national government should have the global responsibility to guaranteeing uniform levels of quality care over the country and overcome local institutions when necessary.

## Background

Rare diseases are life-threatening or chronically debilitating diseases which have an enormous impact on the lives of patients and their families [1]. Researchers have estimated that there are about 7000 different types of rare diseases affecting 350 million people worldwide. Although they affect a small portion of the entire population, they represent a significant societal burden in terms of high morbidity costs and health outcomes.

Among the different types of rare conditions, congenital bleeding disorders (CBD) are one of the most expensive diseases requiring complex and specialized treatments and a high intensity of care. The most common CBD include Hemophilia A, Hemophilia B and the Von Willebrand Disease, which all together represent 95–97% of inherited coagulation deficiencies [2].

Estimates in the US report that there are approximately 20,000 people with hemophilia and that the average healthcare costs per patient are approximately $100,000 to $150,000 per year [3]. The same trend can be found in Europe where hemophilia affects less than 5 people out of 10,000 and where the annual incidence of Hemophilia A is approximately one case per 10,000 births [4].

In recent years, the availability of blood clotting factor concentrates and recombinant technology products have increased the life expectancy of CBD patients and made it comparable to the overall population. Nevertheless, an increase in life expectancy exposes hemophiliac patients to the risk of developing concomitant morbidities and complications. An example is the appearance of inhibitors, which makes replacement therapy ineffective and causes higher risks of physical disability and mortality.

In addition, the management of CBD patients requires appropriate treatments and timely access to integrated care, which should be delivered in specialized treatment centers. These centers are able to meet the various healthcare needs of CBD patients with a multidisciplinary approach that includes specialists from different clinical and non-clinical fields (e.g. specialists in hematology, orthopedics, dentistry, surgery, nurses, physiotherapists, social workers, and allied health professionals). The European guidelines for CBD set standards and criteria for national networks of centers which include number of patients treated (especially if severe) and geographic proximities since caseload by center and timely access to care are two important determinants of care quality for these patients. In particular, small centers (at least 10 severe patients) in these care networks should work in collaboration with Comprehensive Care Centers (at least 40 severe patients) by sharing treatment and diagnostic protocols in order to ensure high standards of care and equity of treatment across geographic areas [5].

In Italy, following the 1993 decentralization reform of the National Health Service (Legislative Decrees 502/1992 and 517/1993) in which several administrative and organizational responsibilities were transferred to the 21 regional administrations, each region provides and administers its services almost autonomously. This is also true for the organization of services to CBD patients, for which, in alignment with the required standards, each regional network should deliver specialized and multidisciplinary, quality care to patients [6, 7].

The Italian National Health Service (NHS), which is founded on the principles of universal coverage to all citizens, is now facing the challenge of assuring uniform quality care levels and timely access to treatment for CBD patients, while containing disproportionately high costs for CBD care with limited resources.

To address these challenges, the Italian Ministry of Health established 54 Hemophilia Treatment Centers (HTCs) for patients with CBD throughout Italy’s regions in 2001. These centers are under the responsibility of each regional administration and they are cost exempt at the local level.

Given these premises, the present article has two main purposes. First, to provide a descriptive overview of the organization of care services for CBD patients across Italian regions. Second, we report the caseloads per HTC, as one of the European standard and criterion for the certification of HTCs and describe interregional mobility of CBD patients as potential measures of the ability of the regional HTC models to provide responsive care to their residents with CBDs [5, 8].

We conclude with some final considerations to stimulate debate among readers and policy makers regarding the organization of care for patients with complex needs when examining the effects of decentralization policies.

## Data and methods

To the best of our knowledge, there is a lack of routinely collected and analyzed data for an in-depth comparative analysis of the performance of Italian HTC centers and their ability to deliver timely and quality care. Available research data and reports on the activities of Italian HTCs are mostly focused on the epidemiology of CBD and the clinical management of CBD patients by using local and national registries, including the national registry of congenital bleeding disorders of the Italian Association of Hemophilia Centers (Associazione Italiana Centri Emofilia-AICE) [9–11]. Since 2003, the AICE collects epidemiological data on prevalence of different congenital bleeding disorders, therapy complications, and drug therapy needs in 51 out of the 54 Italian HTCs and it is currently the only data source available for benchmarking analysis across HTC centers [12]. Unfortunately, the data on costs have been collected for usually one center and not for benchmarking across centers [13]. On the other hand, administrative hospital data sources are increasingly being used to compare service utilization (i.e. inpatients and outpatient visits), processes and outcomes (including mortality, readmissions and complications), between care providers at both the national and international level [14]. However, due to privacy reasons, the link between the different national administrative databases at the individual level is still not completely feasible. Indeed, it is currently not possible to link the national administrative databases with the AICE registry in order to routinely collect standardized clinical and non-clinical information nationwide which would trace the complete care path and service use within and outside the HTCs of CBD patients.

Given these premises, the present study concentrated on providing a picture of the heterogeneity of Italian centers based on the 2012 AICE registry data regarding patient volumes for each HTC center and interregional mobility as potential determinants of the standardization of quality care and responsiveness of the regional network of centers [5, 8]. Volumes were defined as the yearly caseload of patients registered and treated in each center, independently from where they live. Whereas, interregional mobility was measured by comparing each region in terms of percentage of residents who received care within their region of residence and, for the same region, the percentage of the volume of activity generated by residents of other regions (i.e. patients with CBDs who are registered as patients in HTCs of a region which is not their region of residence).

## Results

The 2012 AICE database collected 9946 patient records of 51 out of 54 Italian HTCs (a patient record is a record of the personal and clinical information of patients registered in each HTC). Of these 9946 records, 8362 were of patients treated at only one HTC, whereas the remaining 1584 records regarded the number of “duplicates”, which correspond to 772 patients who were treated at more than one HTC. Consequently, 9134 residents received care in at least one of the Italian HTCs in 2012. Moreover, 2643 of the 9946 patient records (26.5%) were classified as severe cases (those suffering from severe forms of Hemophilia A or B or from Von Willebrand Disease Type 3).

**Fig. 1:**
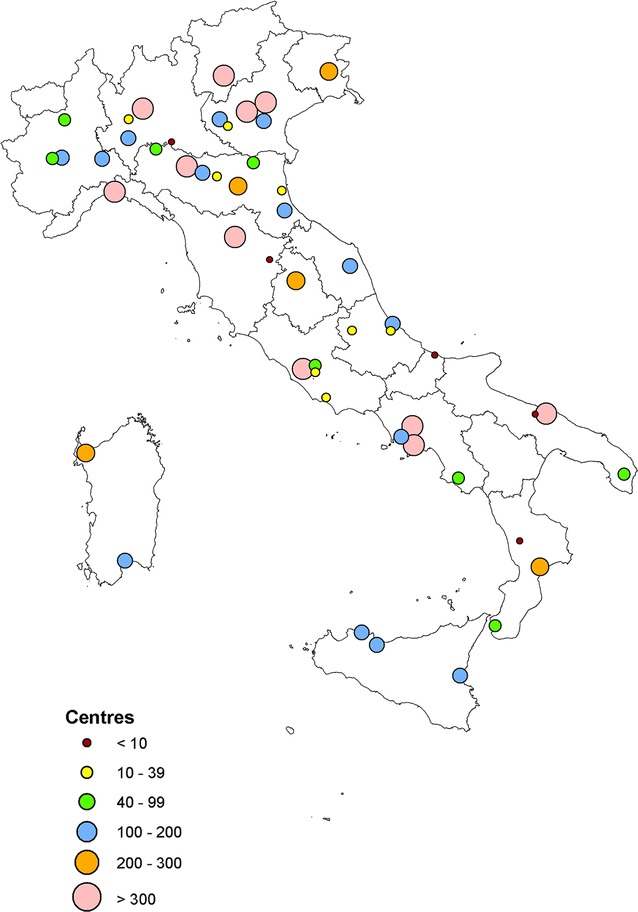
Italian HTCs by geographic location and volumes of patients, year 2012. The figure is created by the authors and not taken from other sources

Figure 1 show the locations of the HTCs throughout the country and the volumes of CBD patients. Moreover, these same data are reported in more detail in Table 1 in which, the first three columns describe the regional context in terms of population size, area in square kilometers and orography where “mountain” stands for more than 60% of the population living in mountainous areas. The last 5 columns of the Table show the number of HTCs per region and their volumes as overall number of patients registered in each center and the number of centers stratified by the number of severe patients treated.

**Table 1:** Distribution of hemophiliac patients enrolled in the Italian HTCs per region

Regions	Population	Area (km^2^)	Orography	No. of centers	Total no. of enrolled patients	Centers with <=10 severe patients	Centers with 11–39 severe patients	Centers with >40 severe patients
Valle D’Aosta	126,620	3261	Mountain	–	–	–	–	–
Molise	313,145	4461	Mountain	1	7	1	–	0
Basilicata	577,562	10,073	Mountain	–	–	–	–	–
Umbria	883,215	8464	Mountain	1	262	0	1	0
Trentino-Alto Adige	1,029,585	13,606	Mountain	1	334	0	1	0
Friuli-Venezia Giulia	1,217,780	7862		1	296	0	1	0
Abruzzo	1,306,416	10,832		3	152	1	2	0
Marche	1,540,688	9401		1	135	0	1	0
Liguria	1,567,339	5,416		1	352	0	0	1
Sardinia	1,637,846	24,100	Island	2	317	0	2	0
Calabria	1,958,418	15,222		3	269	1	2	0
Tuscany	3,667,780	22,987		2	1135	1	–	1
Apulia	4,050,072	19,541		3	718	1	1	1
Emilia-Romagna	4,341,240	22,453		8	1023	5	2	1
Piedmont	4,357,663	25,387		4	448	2	1	1
Veneto	4,853,657	18,407		5	977	1	1	3
Sicily	4,999,854	25,832	Island	3	474	0	1	2
Lazio	5,500,022	17,232		4	980	1	2	1
Campania	5,764,424	13,671		4	938	0	2	2
Lombardy	9,700,881	23,864		4	1129	2	1	1
Total	58,690,025	301,340		51	9946	16	21	14

Both Fig. 1 and Table 1 show a significant geographic variation in terms of volumes of activity and organizational characteristics. In 2012, 16 out of 51 (31.4%) HTCs at the national level treated a number of patients that was under the minimum number required for accreditation (less than 10 severe patients).

Depending on the size of the population, we can distinguish three main groups of regions with different organizational strategies. The first group has small regions (Valle D’Aosta, Molise and Basilicata) with fewer than 600,000 inhabitants; the second group has middle-sized regions (Umbria, Trentino, Friuli Venezia Giulia, Abruzzo, Marche, Liguria, Sardinia and Calabria) with 0.8–1.9 million inhabitants and the third group has large regions (Tuscany, Apulia, Emilia Romagna, Piedmont, Veneto, Sicily, Lazio, Campania and Lombardy) with 3.5 million inhabitants. Given the similar number of inhabitants and assuming a comparable prevalence of CBD patients (the available data on the prevalence of CBD in Italy are 6.2 per 100,000 inhabitants for hemophilia A, 1.2 per 100,000 inhabitants for hemophilia B and 3.7 per 100,000 inhabitants for Von Willebrand disease [7]), regions belonging to the same group should ideally organize their care delivery in the same way. Regions belonging to Group 1 do not have enough CBD patients to create their own HTC and should refer their patients to neighboring regions. This is true for Valle D’Aosta and Basilicata whereas Molise appears to have a center which can treat only seven patients. On the contrary, the large regions belonging to Group 3 have a number of CBD patients which is high enough to require a network of HTCs. There seems to be a significant service organization variability in the regions belonging to this group. For example, in two similar regions, Tuscany and Emilia, Tuscany has centralized its care delivery in two HTC centers (one of which has a number of severe patients under the minimum requirement) whereas Emilia has a network of eight centers with a “hub and spoke” strategy. Although the Lazio and Campania regions have almost the same number of registered patients, they have opted for different organizational strategies. Lazio has centralized most of its patient volumes in one big center (850 patients) and distributed the rest of its patients in three satellite centers. Campania has opted for two big centers (about 300 patients each) and two intermediate centers (about 150 patients each) and one small center.

A significant variability of healthcare delivery models is also registered in the group of medium-sized regions. Regions like Liguria, Sardinia, Calabria and Abruzzo each have a number of severe patients that is sufficiently high (>40) to create one HTC. However, only Liguria has opted for centralizing all its CBD patients into one HTC. On the contrary, the Calabria and Abruzzo regions have distributed their patients in three HTCs and registered a number of severe patients under the standard of a Comprehensive Care Center. Finally, the Sardinia region (differently from Liguria) has allocated its patients to two small centers. However, Sardinia deserves a separate discussion because it is a large island that is considerably far from the rest of Italy.

**Fig. 2:**
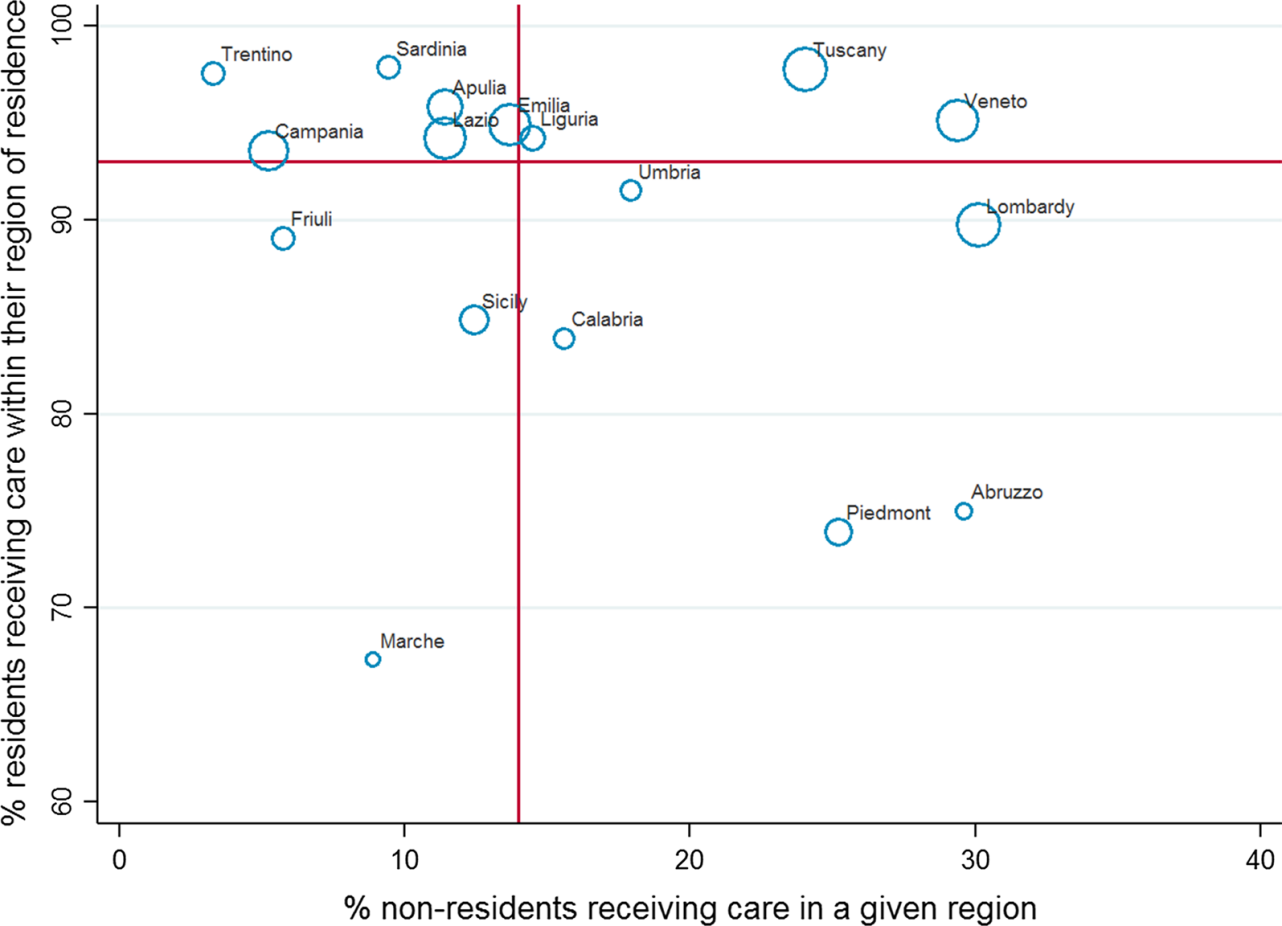
Interregional patient mobility, year 2012. The figure is created by the authors and not taken from other sources

The different organizational strategies adopted by Italy’s regions might have a significant influence on interregional patient mobility (i.e. the extent of services used by residents of a region, whether they receive care in or outside their own region). Figure 2 1 maps each region with the percentage of residents who receive care within their region and with the percentage of the volume of activity generated by residents of other regions. The bubble size is scaled according to the total volume of activity per region and the axes are at the median values.

The evidence from Fig. 2 identifies the clusters of regions. There is an evident concentration of regions in the upper-left quadrant. This means that these regions are characterized by “closed” networks of HTCs in which a small percentage of residents seek care outside their region (10%) and, at the same time, volumes of activity due to incoming patients from other regions is a limited phenomenon (less than 15%). This phenomenon can have several explanations which may depend either on geographical reasons, like the island of Sardinia or organizational reasons, like Emilia Romagna, where residents can meet their needs in their own region. On the contrary, Tuscany and Veneto, which are in the upper right quadrant, have HTCs with a considerable volume of registered patients and they are placed in an excellent position in terms of ability to satisfy their own residents’ needs and in volume of activity from outside the region (more than 20%). This “outside interest” might be explained by the size and specializations of large hospitals where HTCs are located (in particular, surgical and orthopedic specializations). These results, along with the data showing that there are still centers operating under the minimum requirement, suggest that Tuscany and Veneto should reconsider the distribution of HTCs and concentrate their care in highly specialized hospitals which already assure high volumes.

The Abruzzo and Piedmont regions seem to behave differently from the other regions as can be seen in the upper quadrants. In fact, they are characterized by an imbalance between the low percentage of hospital stays by residents (less than 80%) and the high volume of activity due to outside patients (about 30%). According to the data, Abruzzo and Piedmont should be considered part of a larger area together with neighboring regions. Finally, the Marche region does not retain its own residents and it does not attract activity from other regions. Calabria and Sicily have many residents who choose to be treated by a HTC outside their region. This pattern is even more worrying for the island of Sicily which, given its geographic characteristics, should be able to provide timely response to its residents with CBDs.

## Discussions

Rare diseases are a public health problem. Despite their rarity, there are more than 60 million people affected by them in Europe and the US alone. These patients have both medical and social challenges to face as they require prompt access to complex and integrated care from multidisciplinary teams of professionals and they often need life-long access to expensive treatments. Despite the advances in healthcare and the many efforts to develop guidelines for quality standards of care in recent years, the lack of clear pathways and coordination among healthcare stakeholders, as well as organizational gaps in healthcare delivery, still exist [15–18].

In Italy, following the devolution policies of 1990s, regions are free to adopt strategies and models for care provisions, which might differ from one another, but at the same time they should guarantee the same quality care level [8]. Previous studies have demonstrated that these policies have contributed to accentuate interregional disparities in healthcare especially between the northern and the southern regions, which have always been very different for geographical and historical reasons [8, 19]. In this context, our preliminary results are a step toward determining whether leaving the responsibility for rare disease management, such as CBD, to the regions as for the other healthcare services, assure that the regional populations with CBDs receive timely access to comprehensive, specialized treatments in accordance with the existing guidelines everywhere across the country. Although CBD patients might benefit from a regionalized care organization especially in terms of timely access to treatment (which may ultimately be a matter of life and death), these results seem to confirm that the different regional care models for CBDs are still not meeting the required standard of minimum volumes and differ in terms of capacity to retain their residents with CBDs of attract patients from the outside. Indeed, not all the Italian regions have opted for a delivery model in line with the accreditation criteria on volumes. There are regions where HTCs operate below the minimum volume required, and other regions, with similar demographic and geographic characteristics, that have opted for centralizing all their patients in a few high-volume centers. The interregional gap is more evident if we analyze data on patients’ mobility, which might suggest that some regions are not able to retain their patients, presumably because their needs are not completely satisfied by their regional HTC and/or because they expect to receive better care in other regions.

Given these considerable regional disparities, Italy’s national government, whose NHS is responsible for uniform and essential levels of health services across the country, should adopt strategies to bridge the interregional gap in the quality of services provided to CBD patients. Low-performing regions (i.e. regions that do not guarantee the required volumes and have high percentages of residents seeking treatments outside) should be closely monitored by the central government and they should be compelled to find organizational solutions such as agreements with other regions to guarantee that all residents receive prompt access to high quality care.

The main limitation to our study was the lack of comparable individual data at the national level which is widely recognized as one of the main challenge of rare diseases [20, 21], and the lack of data regarding HTC organizational infrastructure including staffing, resources and other organizational components which might influence both efficiency and quality. In Italy, the use of the AICE register alone does not allow record linkage with other medical records. Investments to improve the AICE Register and the centralization of patient information are crucial. Efforts should also be made by the regional governments toward the use of multiple sources of data-by-data linkage at the individual level in order to comprehensively approach patient healthcare paths.

## Conclusions

Preliminary results of the present study show significant disparities among Italian regions in the delivery of care to CBDs patients which could be partly explained by the regional governments that are free to adopt strategies and organization models differing from one region to the other. In this context, this study is committed to stimulating a debate on the importance of targeted service delivery strategies for CBD patients at regional level. Regions alone should not be responsible for the organization and function of HTCs; indeed, intervention measures, among which the systematic monitoring of the performance of the HTCs, should be adopted by the central government to support the implementation of the existing standards for the management of CBDs uniformly across regions.

## Abbreviations

CBDs: congenital bleeding disorders
HTCs: Haemophilia Treatment Centres
NHS: National Health Service
AICE: Associazione Italiana Centri Emofilia

## Footnotes

1 Valle d’Aosta, Basilicata and Molise are not included in the map because the first two regions not have centers and the third region has a not significant volume of activity.
